# Carotid Atherosclerosis, Cerebrospinal Fluid Pressure, and Retinal Vessel Diameters: The Asymptomatic Polyvascular Abnormalities in Community Study

**DOI:** 10.1371/journal.pone.0166993

**Published:** 2016-12-01

**Authors:** Jing Yan Yang, Xuan Yang, Yang Li, Jie Xu, Yong Zhou, An Xin Wang, Xiang Gao, Liang Xu, Shou Ling Wu, Wen Bin Wei, Xing Quan Zhao, Jost B. Jonas

**Affiliations:** 1 Beijing Tongren Eye Center, Beijing Tongren Hospital, Beijing Ophthalmology and Visual Science Key Laboratory, Capital Medical University, Beijing, China; 2 Department of Neurology, Beijing TianTan Hospital, Capital Medical University, Beijing, China; 3 Channing Laboratory, Department of Medicine, Brigham and Women’s Hospital, and Harvard Medical School, Boston, Massachusetts, United States of America; 4 Department of Nutritional Sciences, The Pennsylvania State University, Department of Nutritional Sciences, University Park, Pennsylvania, United States of America; 5 Beijing Institute of Ophthalmology, Ophthalmology and Visual Science Key Lab, Beijing Tongren Eye Center, Beijing Tongren Hospital, Capital Medical University, Beijing, China; 6 Department of Cardiology, Kailuan Hospital, Hebei United University, Tangshan, China; 7 Department of Ophthalmology, Medical Faculty Mannheim of the Ruprecht- Karls-University, Mannheim, Germany; Medical University Innsbruck, AUSTRIA

## Abstract

**Purpose:**

To assess relationships between carotid artery atherosclerosis and retinal arteriolar and venular diameters.

**Methods:**

The community-based longitudinal Asymptomatic Polyvascular Abnormalities Community Study (APAC) included a sub-population of the Kailuan study which consisted of 101,510 employees and retirees of a coal mining industry. Based on the Chinese National Census 2010 and excluding individuals with history of cerebrovascular ischemic events, 4004 individuals were included into the APAC. All participants underwent a detailed clinical examination including blood laboratory tests and carotid artery duplex ultrasound examination. The cerebrospinal fluid pressure (CSFP) was estimated using the formula: CSFP[mmHg] = 0.44xBody Mass Index[kg/m^2^]+0.16xDiastolic Blood Pressure[mmHg]–0.18 x Age[Years]–1.91.

**Results:**

In multivariable analysis (goodness of fit r^2^:0.12), thicker retinal arteries were associated with a thinner common carotid artery intima-media thickness (IMT) (*P* = 0.002; standardized regression coefficient beta:-0.06; non-standardized regression coefficient B:-6.92;95% confidence interval (CI):-11.2,-2.61) after adjusting for thicker retinal nerve fiber layer (*P*<0.001;beta:0.18;B:0.35;95%CI:0.28,0.42), lower diastolic blood pressure (*P*<0.001;beta:-0.16;B:-0.17;95%CI:-0.21,-0.3), younger age (*P*<0.001;beta:-0.08; B:-0.16;95%;CI:-0.25,-0.08), and less abdominal circumference (*P* = 0.003;beta:-0.06;B:-0.11;95%CI:-0.18,-0.03). Thicker retinal vein diameter was associated (r = 0.40) with higher estimated CSFP (*P*<0.001;beta:0.09;B:0.78;95%CI:0.47,1.08) after adjusting for wider retinal arteries (*P*<0.001;beta:0.27;B:0.36;95%CI:0.31,0.41), thicker retinal nerve fiber layer thickness (*P* = 0.03;beta:0.22;B:0.56;95%CI:0.46,0.65) and male gender (*P*<0.001;beta:-0.08;B:-3.98;95%CI:-5.88,2.09).

**Conclusions:**

Thinner retinal artery diameter was significantly, however weakly, associated with increased common carotid artery IMT. It suggests that retinal microvascular changes were only week indicators for an atherosclerotic carotid artery pathology. Thicker retinal vein diameter was associated with higher estimated CSFP, confirming associations between higher estimated CSFP and higher incidence of retinal vein occlusion.

## Introduction

The microvascular system of the retina can be regarded as part of the cerebrovascular system. The central retinal artery is a branch of the ophthalmic artery and indirectly of the internal carotid artery. Using the retinal arterioles as surrogate for the intracranial arterioles may thus offer possibilities for a non-invasive assessment of the cerebrovascular system [1,2]. The central retinal vein drains into the superior ophthalmic vein and eventually into the intracranial cavernous sinus. Changes in the retinal veins may thus reflect changes in the intracranial venous system and, indirectly, changes in the cerebrospinal fluid pressure (CSFP), since the intracranial venous pressure is influenced by the CSFP. Numerous previous studies have revealed relationships between arterial hypertension and retinal microvascular changes, such as arterio-venous crossing signs, retinal arterial thinning and an increased arterio-venous width ratio [1–6]. Other investigations showed correlations between retinal microvascular abnormalities, defects of the retinal nerve fiber layer and an increased prevalence of previous or acute cerebral strokes [1,2,7]. Studies on the relationship between the venous retinal system and intracranial parameters and investigations on the relationship between atherosclerotic changes in the cerebroafferent arteries and the retinal arterial system, however, have been scarce so far [8]. We therefore conducted this study to explore potential associations between atherosclerotic changes in the cerebroafferent arteries and the retinal arterial diameter as well as associations between the retinal vein diameter and parameters influencing the CSFP. We chose the design of a community-based study population which had routinely undergone sonographic examination of the carotid arteries independently of the presence of cerebrovascular symptoms.

## Methods

The Asymptomatic Polyvascular Abnormalities Community study (APAC) is a community-based observational study which examined the epidemiology of asymptomatic polyvascular abnormalities in Chinese adults [9,10]. The APAC has been described in detail recently [9,10]. The study cohort was a sub-population of the population of the Kailuan study which consisted of 101,510 employees and retirees of the Kailuan (Group) Co. Ltd, a large coal mining industry in Tangshan. The city of Tangshan is located 150 km southeast of Beijing in the province of Hebei. Based on the data of the Chinese National Census from 2010 and using a stratified random sampling method by year of age and gender, we randomly selected a sample of 7000 subjects with an age of 40+ years out of the Kailuan cohort. A total of 5,852 individuals agreed to participate in the APAC study and 5,816 individuals completed the baseline data collection. The inclusion criteria of the present study (no history of stroke, transient ischemic attack, and coronary disease at baseline; and absence of neurologic deficits indicating previous stroke) were not met by 376 individuals. Out of the remaining 5,440 participants, 4004 individuals underwent ocular fundus photography and optical coherence tomography of the optic nerve head in the period from 2012 to 2014 and were eventually included into our study. All study participants underwent a detailed clinical examination with blood laboratory tests and carotid artery duplex ultrasound examination. The study was approved by the Ethics Committees of the Kailuan General Hospital and the Beijing Tiantan Hospital according to the guidelines of the Helsinki Declaration. All participants gave their written informed consent.

As described in detail previously [9], the interview performed by trained investigators consisted of a standardized questionnaire with questions on the demographic and socioeconomic background, educational level, history of major diseases, alcohol consumption and smoking. We measured body height and weight and calculated the body mass index (BMI). Smoking was defined as smoking at least one cigarette per day for more than a year. Alcohol consumption included the intake of at least 80g of liquor a day for more than one year. A systolic blood pressure of <120 mmHg and a diastolic pressure <80 mmHg were considered to be normal. A systolic pressure of 120 mmHg to 139 mmHg or a diastolic pressure of 80 mmHg to 89 mmHg were defined as prehypertension, and a systolic pressure ≥140 mmHg or a diastolic pressure of ≥90 mmHg were defined as arterial hypertension. If the systolic measurements and diastolic values fell into different categories, the study participant was assigned to the higher category. Intake of anti-hypertension medication was categorized as arterial hypertension.

Each study participant underwent a bilateral carotid artery duplex sonography and we determined the intima-media thickness (IMT). According to the recommendations published by the Society of Radiologists in Ultrasound Consensus Conference, a carotid artery stenosis (≥50%) was categorized into several grades [11]. Blood concentrations of glucose and blood lipids were measured in blood samples which were collected from the antecubital vein in the morning after an overnight fasting.

As described and applied in previous population-based studies, all study participants underwent ocular fundus photography (non-mydriatic digital fundus camera Type CR6-45NM; Canon, Ōta, Tokyo, Japan). Simultaneous stereoscopic 45°color fundus photographs centered on the optic disc (Diabetic Retinopathy Study standard field 1) and on the macula (Diabetic Retinopathy Study standard field 2) were obtained from each eye. Using a computer assisted quantitative assessment software (IVAN; University of Wisconsin, Madison, WI), we determined the retinal vessel diameters in the right eyes of each study participant, so that the data of only one eye per individual were taken for the statistical analysis. The average retinal arteriolar or venular width was measured applying the Big–6 formula. It was presented as the central retinal arteriolar equivalent or central retinal venular equivalent. The arteriovenous ratio was calculated as central retinal venular equivalent divided by the central retinal arteriolar equivalent. The method has been described in detail in previous population-based studies [12]. The reproducibility of the retinal vessel diameter determinations by the computer assisted quantitative assessment was, as expressed as relative coefficient, 0.97 for the intra-observer variability and 0.92 for the inter-observer variability [13].

We used a commercially available software program (SPSS software, version 22.0, IBM-SPSS, Chicago, USA) for the statistical analysis. Continuous variables were described by their means ± standard deviations and categorical variables were presented as percentages. The main outcome parameters of the study were the retinal vessel diameters. Only one eye per study participant was included into the statistical analysis. In a first step of the analysis, we performed a univariate analysis of the associations between the retinal vessel diameters and other ocular and systemic variables. In a second step, we conducted a multivariable linear regression analysis, with the retinal vessel diameters as dependent variable and all those parameters as independent variables which were associated with the retinal vessel diameters in the univariate analysis. The cut-off value for the *P*-value was <0.10. From the list of independent parameters we then dropped step by step those parameters which either showed a high collinearity or which were no longer significantly associated with the outcome parameter. The cut-off value for high collinearity was a variance inflation factor (VIF) of higher than 2. We finally repeated the multivariable analysis in a reverse manner, with artery IMT as dependent variable and retinal artery diameter as independent variables after adjusting for other parameters such as age, gender, blood pressure, level of education and concentration of high-density lipoproteins and low-density lipoproteins. We calculated the standardized regression coefficient beta and non-standardized regression B and the 95% confidence intervals (CIs) of B. In the multivariable linear regression analysis, a *P*-value smaller than 0.05. was considered to be statistically significant.

## Results

**Fig 1:**
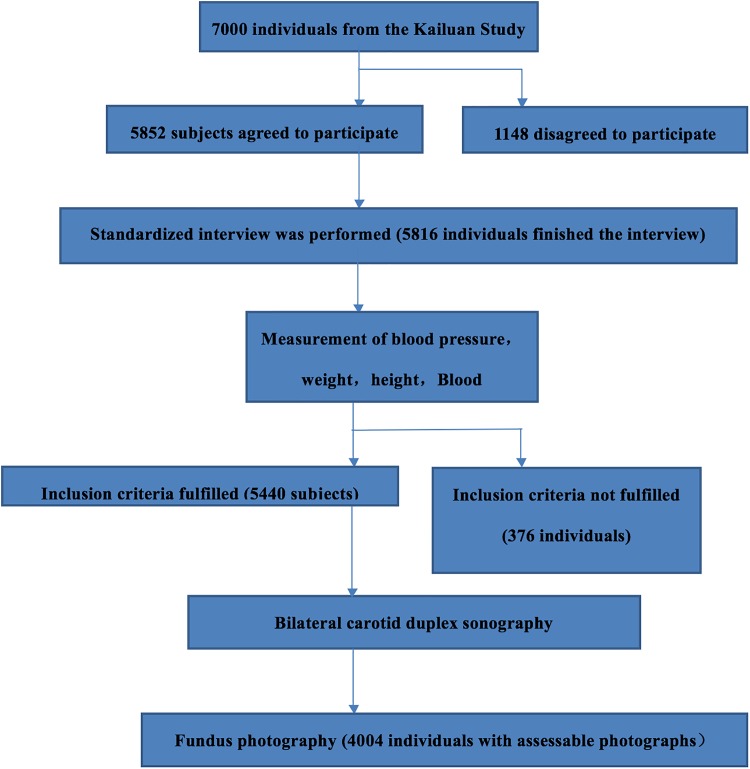
Flowchart Describing the Recruitment of Study Participants.

The study included 4004 participants (1720 (43.0%) women) with a mean age of 59.7 ± 11.0 years (Table 1) (Fig 1). The study participants and the study non-participants differed significantly in age (59.7 ± 11.0 years versus 55.4 ± 13.5 years; *P*<0.001), gender (woman/men: 43%/57% versus 68.3%/31.7%; *P*<0.001), abdomen circumference (92.38 ± 10.25 cm versus 88.84 ± 10.30 cm; *P*<0.001), hip circumference (99.33 ± 9.77 cm versus 97.27± 8.51 cm; *P*<0.001), and level of education (3.4 ± 0.8 versus 3.2 ± 0.8; *P*<0.001). There was no significant difference in mean arterial pressure (98.72 ± 13.04 mm Hg versus 98.05 ± 13.38 mm Hg; *P* = 0.10), body mass index (24.90 ± 3.41 kg/m2 versus 24.9 ± 3.52 kg/m2; *P* = 0.93), waist circumference (86.3 ± 16.2 cm versus 86.7 ± 9.9 cm; *P* = 0.32). The mean retinal arteriolar and venular calibers were 153.6 ± 19.77 μm and 232.14± 26.13 μm, respectively.

**Table 1:** Characteristics (Mean ± Standard Deviation) of the Study Populations.

Parameters	Patients (n = 4004)
Age	59.7 ± 11.0
Women/ Men	1720 / 2284 (43% / 57%)
Triglyceride (mmol/L)	1.70 ± 2.16
Fasting Blood Glucose (mmol/L)	5.74 ± 1.72
Waist Circumference (cm)	86.29 ± 16.16
Abdomen Circumference (cm)	92.38 ± 10.25
Hip Circumference (cm)	99.33 ± 9.77
Mean Arterial Pressure (mmHg)	98.72±13.04
Body Mass Index (kg/m^2^)	24.90 ± 3.41
Common Carotid Artery Intima-Media Thickness (mm) Right	0.80 ± 0.18
Common Carotid Artery Intima-Media Thickness (mm) Left	0.81 ± 0.19
Internal Carotid Artery Intima-Media Thickness (mm) Right	0.52 ± 0.18
Internal Carotid Artery Intima-Media Thickness (mm) Left	0.52 ± 0.18
Initial Segment of Subclavian Artery Intima-Media Thickness (mm) Right	0.97 ± 0.20
Initial Segment of Subclavian Artery Intima-Media Thickness (mm) Left	0.85 ± 0.18
Retinal Arteriolar Caliber (μm)	153.6 ± 19.8
Retinal Venular Caliber (μm)	232.1 ± 26.1
Arterio-Venous Ratio	0.67 ± 0.11

**Fig 2:**
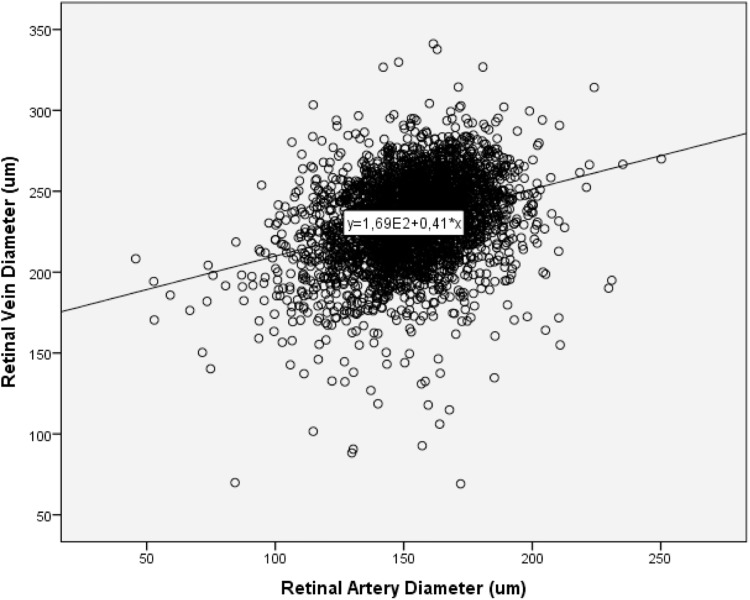
Scattergram Showing the Association between Retinal Artery Diameter and Retinal Venous Diameter (*P*<0.001; regression coefficient: 0.31; equation of the regression line: Arterial Diameter (μm) = 0.24 x Venous Diameter (μm) + 98.6).

**Fig 3:**
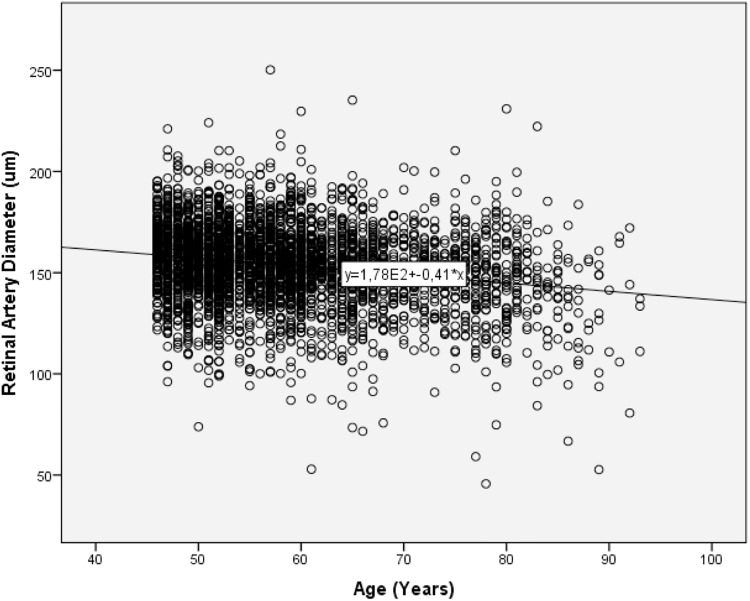
Scattergram Showing the Association between Retinal Artery Diameter and Age (*P*<0.001; regression coefficient: -0.21; equation of the regression line: Arterial Diameter (μm) = -0.41 x Age (Years) + 177).

In univariate analysis, retinal artery diameter increased significantly with thicker retinal vein diameter (*P*<0.001; equation of the regression line: Arterial Diameter (μm) = 0.24 x Venous Diameter (μm) + 98) (Fig 2). Retinal artery diameter decreased significantly with older age (*P*<0.001; equation of the regression line: Arterial Diameter (μm) = -0.41 x Age (Years) + 178) (Fig 3).

Since many parameters such as results of the biochemical blood analysis and blood pressure were dependent on age, we primarily adjusting them for age and then assessed their association with the retinal vessel diameters (Table 2). After adjustment for age, wider retinal arteries were significantly associated with female gender (*P*<0.001), lower body mass index (*P*<0.001), lower hip and abdomen circumference (*P*<0.001), lower frequency of alcohol consumption (*P* = 0.001), lower systolic, diastolic and mean blood pressure (*P*<0.001), lower prevalence of anti-hypertensive therapy (*P*<0.001), lower estimated cerebrospinal fluid pressure (*P*<0.001), lower prevalence of fatty liver disease (*P*<0.001), thinner common carotid artery IMT (left and right) (*P* = 0.001), slower pulse wave velocity at the upper arm (left and right) (*P*<0.001), lower blood concentration of uric acid (*P* = 0.004), larger neuroretinal rim area (*P*<0.001) and volume (*P* = 0.007), thicker retinal nerve fiber layer (*P*<0.001), and lower vertical cup/disc diameter ratio (*P* = 0.01) (Table 2). There was a statistically marginal association between wider retinal arteries and lower frequency of drinking tea (*P* = 0.05) and lower blood concentrations of triglycerides (*P* = 0.07) and total bilirubin (*P* = 0.06) and thinner full retinal fovea thickness (P = 0.04) (Table 2).

**Table 2:** Associations (Univariate Analysis) between Retinal Artery Diameter (μm) and Ocular and General Parameters After Adjusting for Age.

Characteristics	*P*-Value	Standardized Regression Coefficient beta	Non-Standardized Regression Coefficient B	95% Confidence Interval of B	Number of Individuals Included in the Analysis
Gender	<0.001	0.11	4.52	3.23, 5.81	4004
Body Mass Index (kg/m^2^)	<0.001	-0.66	-0.38	‘-0.58, -0.19	3602
Abdomen Circumference (cm)	<0.001	-0.09	-0.18	-0.25, -0.12	3595
Hip Circumference (cm)	<0.001	-0.07	-0.15	-0.22, -0.08	3390
Marriage Status	0.92	N	N	N	3309
Level of Education	0.23	N	N	N	3248
Frequency of Physical Exercise (Never/Sometimes/Often)	0.22	N	N	N	3331
Smoking (Never/Former/Current)	0.20	N	N	N	3911
Smoking, Passive	0.21	N	N	N	1169
Alcohol Consumption Frequency	0.001	-0.06	-0.72	-1.13, -0.31	3927
Salt Intake	0.20	N	N	N	3906
Tea Drinking Frequency	0.05	-0.03	-0.29	-0.59, 0.01	3937
Snoring (Intensity 1–3)	0.20	N	N	N	3331
Systolic Blood Pressure (mm Hg)	<0.001	-0.18	-0.18	-0.22, -0.15	3741
Diastolic Blood Pressure (mm Hg)	<0.001	-0.16	-0.26	-0.32, -0.21	3739
Mean Arterial Pressure (mmHg)	<0.001	-0.18	-0.27	-0.32, -0.22	3738
Hypertension Family History	0.037	-0.04	-2.32	-4.50, -0.141	2868
Use of Anti-Hypertensive Medication	0.001	-0.06	-1.7	-2.66, -0.74	3317
Heart Rate	0.50	N	N	N	3603
Heart Infarct History	0.90	N	N	N	3313
Cardiac Premature Beats	0.66	N	N	N	3149
Atrial Flutter	0.81	N	N	N	3149
Estimated Cerebrospinal Fluid Pressure (mm Hg)	<0.001	-0.17	-1.08	-1.33, -0.83	3368
Fatty Liver Disease	<0.001	-0.08	-1.8	-2.65,-0.94	3212
Common Carotid Artery Intima-Media Thickness (mm) Right	0.001	-0.06	-6.83	-10.68,-2.99	3953
Common Carotid Artery Intima-Media Thickness (mm) Left	0.001	-0.06	-6.47	-10.18,-2.77	3954
Common Carotid Artery Stenosis Right	0.35	N	N	N	3687
Common Carotid Artery Stenosis Left	0.76	N	N	N	3689
Internal Carotid Artery Intima-Media Thickness (mm) Right	0.11	N	N	N	3944
Internal Carotid Artery Intima-Media Thickness (mm) Left	0.02	-0.04	-4.25	-7.88,-0.62	3944
Internal Carotid Artery Stenosis Right	0.33	N	N	N	3657
Internal Carotid Artery Stenosis Left	0.70	N	N	N	3648
Initial Segment of Subclavian Artery Intima-Media Thickness (mm) Right	0.35	N	N	N	3902
Initial Segment of Subclavian Artery Intima-Media Thickness (mm) Left	0.005	-0.05	-5.47	-9.26,-1.68	3877
Initial Segment of Subclavian Artery Stenosis Right	0.83	N	N	N	3637
Initial Segment of Subclavian Artery Stenosis Left	0.62	0.03	1.82	-0.52, 4.16	3553
Vertebral Artery Intima-Media Thickness (mm) Right	0.93	N	N	N	273
Vertebral Artery Intima-Media Thickness (mm) Left	0.95	N	N	N	273
Vertebral Artery Stenosis Right	0.49	N	N	N	2018
Vertebral Artery Stenosis Left	0.49	N	N	N	2017
Ankle-Brachial Index (Right)	0.11	N	N	N	3764
Ankle-Brachial Index (Left)	0.41	N	N	N	3764
Pulse Wave Velocity Upper Right Arm	<0.001	-0.10	-0.003	-0.005, -0.002	3764
Pulse Wave Velocity Upper Left Arm	<0.001	-0.10	-0.003	-0.005, 0.002	3764
Diabetes mellitus, History of	0.88	N	N	N	3330
Diabetes Mellitus Type	0.19	N	N	N	247
Use of Anti-Diabetic Medication	0.36	N	N	N	239
Hyperlipidemia, History of	0.47	N	N	N	3330
Cerebral Stroke, History of	0.77	N	N	N	60
Pregnancies, Number of	0.13	N	N	N	1655
Menstruation Duration (Years)	0.45	N	N	N	777
Menarche (Age in Years)	0.82	N	N	N	1646
Menopause (Age in Years)	0.34	N	N	N	777
Hormone Replacement Medication	0.94	N	N	N	1053
Cognitive Function, Mini Mental Test, Total Score	0.36	N	N	N	3260
Cognitive Function, Mini Mental Test, Level of Education	0.53	N	N	N	3383
Cognitive Function, Montreal Test, Total Score	0.47	N	N	N	816
Cognitive Function, Montreal Test, Orientation Score	0.19	N	N	N	796
Blood Concentration Fasting Blood Glucose (mmol/L)	0.31	N	N	N	3199
Blood Concentration Triglycerides (mmol/L)	0.07	-0.03	-0.28	-0.59, -0.02	3377
Blood Concentration High-Density Lipoproteins (mmol)	0.30	N	N	N	3376
Blood Concentration Low-Density Lipoproteins (mmol)	0.72	N	N	N	3375
Blood Concentration Total Cholesterol (mmol)	0.84	N	N	N	3373
Blood Concentration Hemoglobin (mmol)	0.18	N	N	N	3380
Blood White Cell Count	0.12	N	N	N	3380
Blood Concentration High-Sensitive Reactive Protein	0.91	N	N	N	2642
Blood Concentration Creatinine (mmol)	0.34	N	N	N	3376
Blood Concentration Urea (mmol)	0.50	N	N	N	3376
Blood Concentration Total Bilirubin (mmol)	0.06	-0.03	-0.1	-0.2, 0.005	3374
Blood Concentration Direct Bilirubin (mmol)	0.47	N	N	N	2791
Blood Concentration Uric Acid (mmol)	0.004	-0.05	-0.01	-0.02, -0.004	3357
Blood Concentration Total Protein	0.48	N	N	N	2516
Blood Concentration Albumin	0.76	N	N	N	2338
Blood Concentration Glutamic-Pyruvic Transaminase	0.29	N	N	N	3377
Optic Disc Area (mm^2^)	0.13	N	N	N	3376
Neuroretinal Rim Area (mm^2^)	<0.001	0.07	3.18	1.69, 4.68	3376
Neuroretinal Rim Volume (mm^3^)	0.007	0.05	7.88	2.16, 13.6	3376
Retinal Nerve Fiber Layer Thickness Mean (μm)	<0.001	0.20	0.36	0.30, 0.43	3376
Vertical Cup/Disc Diameter ratio	0.01	-0.43	-3.86	-6.94, -0.79	3376
Full Retinal Fovea Thickness Mean (μm)	0.04	-0.04	-0.03	-0.05, -0.001	3407
Retinal Venular Caliber (μm)	<0.001	0.28	0.21	0.19, 0.24	3595
Arterio-Venous Ratio	<0.001	0.6	104.54	99.9, 109	3595

In the following multivariable linear regression due to reasons of collinearity, we dropped step by step the parameters of waist circumference (VIF: 5.5), common carotid artery IMT (left) (VIF: 5.1), and pulse wave velocity upper left arm (VIF: 4.2) and neuroretinal rim volume (VIF: 3.5). Due to missing statistical significance, we then dropped body mass index (*P* = 0.94), tea drinking frequency (*P* = 0.97), estimated cerebrospinal fluid pressure (*P* = 0.94), blood concentration of triglycerides (*P* = 0.85), blood concentration of uric acid (*P* = 0.68), use of anti-hypertensive medication (*P* = 0.62), frequency of alcohol consumption (*P* = 0.76), internal carotid artery IMT(left) (*P* = 0.33), internal carotid artery IMT(right) (*P* = 0.62), fatty liver disease (*P* = 0.35), blood concentration of total bilirubin (*P* = 0.15), vertical optic cup/disc diameter ratio (*P* = 0.07), neuroretinal rim area (right) (*P* = 0.23), diastolic blood pressure (*P* = 0.08), pulse wave velocity upper arm (right) (*P* = 0.07), and female gender (*P* = 0.06).

In the final model (goodness of fit of the multivariable model r^2^: 0.12), thicker retinal arteries were associated with younger age (*P*<0.001), less abdominal circumference (*P* = 0.003), lower diastolic blood pressure (*P*<0.001), thicker retinal nerve fiber layer (*P*<0.001) and thinner IMT of the initial segment of the subclavian artery (*P* = 0.01). If the subclavian artery IMT was replaced by the common carotid artery IMT, a thinner common carotid artery IMT was significantly associated with a thicker retinal artery diameter (*P* = 0.002) (Table 3).

**Table 3:** Associations (Multivariable Analysis) between Retinal Artery Diameter and Ocular and Systemic Parameters.

Characteristics	*P*-Value	Standardized Regression Coefficient beta	Non-Standardized Regression Coefficient B	95% Confidence Interval of B	Variance Inflation Factor
Age (Years)	<0.001	-0.08	-0.16	-0.25, -0.08	1.37
Abdomen Circumference (mm)	0.003	-0.06	-0.11	-0.18, -0.03	1.13
Diastolic Blood Pressure (mm Hg)	<0.001	-0.16	-0.17	-0.21, -0.13	1.27
Retinal Nerve Fiber Layer Thickness (μm)	<0.001	0.18	0.35	0.28, 0.42	1.08
Intima-Media Thickness of the Right Common Carotid Artery (mm)	0.002	-0.06	-6.92	-11.2, -2.61	1.21

In a reverse manner, thicker common artery IMT was associated with thinner retinal artery diameter (*P* = 0.03; B: 0.000; 95%CI: -0.001, 0.000) after adjusting for older age (*P*<0.001; B: 0.005; 95%CI: 0.004, 0.006), male gender (*P* = 0.02; B: -0.02; 95%CI: -0.03, -0.002), higher systolic blood pressure (*P*<0.001; B: 0.001; 95%CI: 0.001, 0.001), lower level of education (*P* = 0.002; B: -0.01; 95%CI: -0.02, -0.01), higher prevalence of fatty liver disease (*P* = 0.02; B: 0.01; 95%CI: 0.001, 0.02), lower concentration of high-density lipoproteins (*P* = 0.002; B: -0.02; 95%CI: -0.03, -0.01) and higher concentration of low-density lipoproteins (*P*<0.001; B: 0.01; 95%CI: 0.02, 0.02).

As for retinal vein diameter, we assessed the association between the retinal vein diameter and other parameters primarily after adjusting for age (Table 4). After adjustment for age, thicker retinal veins were significantly associated with female gender (*P* = 0.01), higher body mass index (*P* = 0.02), higher waist circumference (*P*<0.001), and abdomen circumference (*P* = 0.001), higher frequency of drinking tea (*P* = 0.05), higher prevalence of snoring (*P* = 0.03), lower systolic blood pressure (*P* = 0.02), lower cognitive function score as tested in the mini mental test (level of education) (*P* = 0.02), higher blood concentration of creatinine (*P* = 0.04), larger optic disc area (*P*<0.001), larger neuroretinal rim area (*P*<0.001) and volume (*P*<0.001), thicker retinal nerve fiber layer (*P*<0.001), and lower vertical cup/disc diameter ratio (*P* = 0.001) (Table 4). There was a statistically marginal association between wider retinal veins and marriage status (*P* = 0.05), lower level of education (*P* = 0.08), higher heart rate (*P* = 0.04), faster pulse wave velocity at the upper right arm (*P* = 0.01), higher prevalence of a previous ischemic cerebral stroke (*P* = 0.007), lower cognitive function score as tested in the mini mental test (total score) (*P* = 0.06), lower cognitive function score as tested in the Montréal test (total score: *P* = 0.06; orientation score: *P* = 0.10), lower blood concentration of high-density lipoproteins (*P* = 0.07), higher white cell blood count (*P* = 0.09) and internal carotid artery IMT (right) (*P* = 0.07) (Table 4).

**Table 4:** Associations (Univariate Analysis) Between Retinal Vein Diameter (μm) and Ocular and General Parameters After Adjusting for Age.

Characteristics	*P*-Value	Standardized Regression Coefficient beta	Non-Standardized Regression Coefficient B	95% Confidence Interval of B	Number of Individuals Included in the Analysis
Gender	0.01	-0.04	-2.22	-3.93, -0.51	4004
Body Mass Index (kg/m^2^)	0.02	0.04	0.32	0.6, 0.58	3602
Waist Circumference (cm)	<0.001	0.06	0.1	0.04, 0.16	3897
Abdomen Circumference (cm)	0.001	0.06	0.14	0.06, 0.23	3595
Hip Circumference (cm)	0.01	0.05	0.12	0.03, 0.22	3390
Marriage Status	0.05	-0.04	-3.47	-6.95, 0.01	3309
Level of Education	0.08	-0.03	-1.06	-2.24, 0.13	3248
Frequency of Physical Exercise (Never/Sometimes/Often)	0.44	N	N	N	3331
Alcohol Consumption Frequency	0.17	N	N	N	3927
Salt Intake	0.43	N	N	N	3906
Tea Drinking Frequency	0.05	0.03	0.4	0.01, 0.79	3937
Snoring (Intensity 1–3)	0.03	0.04	1.53	0.15, 2.91	3331
Systolic Blood Pressure (mm Hg)	0.02	-0.18	-0.18	-0.22, -0.15	3741
Diastolic Blood Pressure (mm Hg)	0.72	N	N	N	3739
Mean Arterial Pressure (mmHg)	0.20	N	N	N	3738
Hypertension Family History	0.21	N	N	N	2868
Use of Anti-Hypertensive Medication	0.21	N	N	N	3317
Heart Rate	0.04	0.04	0.04	0.002, 0.07	3603
Heart Infarct History	0.90	N	N	N	3313
Cardiac Premature Beats	0.66	N	N	N	3149
Atrial Flutter	0.92	N	N	N	3149
Estimated Cerebrospinal Fluid Pressure (mm Hg)	0.45	N	N	N	3368
fatty liver disease	0.16	N	N	N	3212
Common Carotid Artery Intima-Media Thickness (mm) Right	0.18	N	N	N	3953
Common Carotid Artery Intima-Media Thickness (mm) Left	0.14	N	N	N	3954
Common Carotid Artery Stenosis Right	0.13	N	N	N	3687
Common Carotid Artery Stenosis Left	0.76	N	N	N	3689
Internal Carotid Artery Intima-Media Thickness (mm) Right	0.07	0.03	4.57	-0.34, 9.47	3944
Internal Carotid Artery Intima-Media Thickness (mm) Left	0.19	N	N	N	3944
Internal Carotid Artery Stenosis Right	0.70	N	N	N	3657
Internal Carotid Artery Stenosis Left	0.49	N	N	N	3648
Initial Segment of Subclavian Artery Intima-Media Thickness (mm) Right	0.58	N	N	N	3902
Initial Segment of Subclavian Artery Intima-Media Thickness (mm) Left	0.37	N	N	N	3877
Initial Segment of Subclavian Artery Stenosis Right	0.14	N	N	N	3637
Initial Segment of Subclavian Artery Stenosis Left	0.53	N	N	N	3553
Vertebral Artery Intima-Media Thickness (mm) Right	0.37	N	N	N	273
Vertebral Artery Intima-Media Thickness (mm) Left	0.38	N	N	N	273
Vertebral Artery Stenosis Right	0.85	N	N	N	2018
Vertebral Artery Stenosis Left	0.85	N	N	N	2017
Ankle-Brachial Index (Right)	0.95	N	N	N	3764
Ankle-Brachial Index (Left)	0.71	N	N	N	3764
Pulse Wave Velocity Upper Right Arm	0.01	-0.05	-0.002	-0.004, 0.000	3764
Pulse Wave Velocity Upper Left Arm	0.04	-0.04	-0.002	-0.003, 0.000	3764
Diabetes mellitus, History of	0.05	0.03	3.64	0.09, 7.19	3330
Diabetes Mellitus Type	0.19	N	N	N	247
Use of Anti-Diabetic Medication	0.45	N	N	N	239
Hyperlipidemia, History of	0.40	N	N	N	3330
Cerebral Stroke, History of	0.007	0.36	44.19	12.3, 76.1	60
Pregnancies, Number of	0.63	N	N	N	1655
Menstruation Duration (Years)	0.65	N	N	N	777
Menarche (Age in Years)	0.25	N	N	N	1646
Menopause (Age in Years)	0.75	N	N	N	777
Hormone Replacement Medication	0.35	N	N	N	1053
Cognitive Function, Mini Mental Test, Total Score	0.06	-0.04	-0.36	-0.72, 0.01	3260
Cognitive Function, Mini Mental Test, Level of Education	0.02	-0.05	-3.16	-0.72, -0.59	3383
Cognitive Function, Montreal Test, Orientation Total Score	0.1	-0.06	-4.79	-10.4, 0.84	796
Cognitive Function, Montreal Test, Total Score	0.06	-0.07	-0.4	-0.80, 0.02	816
Blood Concentration Fasting Blood Glucose (mmol/L)	0.16	N	N	N	3199
Blood Concentration Triglycerides (mmol/L)	0.64	N	N	N	3377
Blood Concentration High-Density Lipoproteins (mmol)	0.07	-0.03	-1.74	-3.62, 0.15	3376
Blood Concentration Low-Density Lipoproteins (mmol)	0.36	N	N	N	3375
Blood Concentration Total Cholesterol (mmol)	0.98	N	N	N	3373
Blood Concentration Hemoglobin (mmol)	0.14	N	N	N	3380
Blood White Cell Count	0.09	0.03	0.38	-0.06, 0.83	3380
Blood Concentration High-Sensitive Reactive Protein	0.38	N	N	N	2642
Blood Concentration Creatinine (mmol)	0,04	0.04	0.03	0.001, 0.05	3376
Blood Concentration Urea (mmol)	0.11	N	N	N	3376
Blood Concentration Total Bilirubin (mmol)	0.48	N	N	N	3374
Blood Concentration Direct Bilirubin (mmol)	0.32	N	N	N	2791
Blood Concentration Uric Acid (mmol)	0.85	N	N	N	3357
Blood Concentration Total Protein	0.80	N	N	N	2516
Blood Concentration Albumin	0.47	N	N	N	2338
Blood Concentration Glutamic-Pyruvic Transaminase	0.31	N	N	N	3377
Optic Disc Area (mm2)	<0.001	0.07	4.4	2.35, 6.44	3376
Neuroretinal Rim Area (mm2)	<0.001	0.11	6.04	4.1, 8.0	3376
Neuroretinal Rim Volume (mm3)	<0.001	0.08	17.87	10.41, 35.33	3376
Retinal Nerve Fiber Layer Thickness Mean (μm)	<0.001	0.26	0.64	0.56, 0.73	3376
Vertical Cup/Disc Diameter ratio	0.001	-0.06	-6.74	-10.75, -2.73	3376
Retinal Arterial Caliber (μm)	<0.001	0.28	0.38	0.33, 0.42	3595
Arterio-Venous Ratio	<0.001	-0.54	-128.92	-135–123	3595

In the multivariable analysis due to reasons of collinearity, we dropped waist circumference (VIP: 12.3), pulse wave velocity upper left arm (VIP: 6.3), abdomen circumference (VIF: 5.4), neuroretinal rim area (VIF: 5.2), and vertical cup/disc ratio (VIF: 3.3). Due to lack of statistical significance, we then dropped mini mental state examination results (*P* = 0.79), Montréal cognitive assessment test orientation total score (*P* = 0.60), mini mental state examination educational level (*P* = 0.34), Montréal cognitive assessment test total score (*P* = 0.14), level of education (*P* = 0.71), status of marriage (*P* = 0.68), white blood cell count (*P* = 0.77), internal carotid artery IMT (right) (*P* = 0.91), hip circumference (*P* = 0.65), pulse wave velocity (*P* = 0.47), optic disc area (*P* = 0.65), blood creatinine concentration (*P* = 0.57), neuroretinal rim volume (*P* = 0.52), stroke history (*P* = 0.35), snoring (*P* = 0.26), high-density lipoproteins concentration (*P* = 0.25), tea drinking frequency (*P* = 0.17), and history of diabetes mellitus (*P* = 0.11).

In the final model (goodness of fit of the multivariable model r^2^: 0.12), thicker retinal veins were associated with younger age (*P*<0.001), female gender (*P*<0.001), higher body mass index (*P* = 0.002), lower systolic blood pressure (*P*<0.001), higher heart rate (*P* = 0.037), and thicker retinal nerve fiber layer (*P*<0.001) (Table 5). If we replaced age, body mass index and systolic blood pressure by the estimated cerebrospinal fluid pressure, thicker retinal vein diameter was significantly associated with the estimated cerebrospinal fluid pressure (*P*<0.001; B: 0.67; 95%CI: 0.35, 0.99) after adjusting for male gender (*P* = 0.03; B: -2.22; 95%CI: -4.18, -0.27), and thicker retinal nerve fiber layer (*P*<0.001; B: 0.70; 95%CI: 0.62, 0.80). Heart rate was no longer significantly (*P* = 0.08) associated with retinal vein diameter in that model. If we added retinal artery diameter to the model, the regression coefficient increased to r = 0.40 and thicker retinal vein diameter was correlated with higher estimated cerebrospinal fluid pressure (*P*<0.001), male gender (*P*<0.001), thicker retinal nerve fiber layer thickness (*P* = 0.03) and wider retinal arteries (*P*<0.001) (Table 6).

**Table 5:** Associations (Multivariable Analysis) between Retinal Vein Diameter and Ocular and Systemic Parameters.

Parameter	*P*-Value	Standardized Regression Coefficient beta	Non- Standardized Regression Coefficient B	95% Confidence Interval of B	Variance Inflation Factor
Age (Years)	<0.001	-0.12	-0.33	-0.43, -0.22	1.27
Gender	<0.001	-0.08	-4.29	-6.25, -2.32	1.08
Body Mass Index (kg/m^2^)	0.002	0.06	0.45	0.17, 0.74	1.08
Systolic Blood Pressure (mm Hg)	<0.001	-0.08	-0.11	-0.17, -0.06	1.32
Heart Rate	0.037	0.04	0.04	0.002, 0.07	1.00
Retinal Nerve Fiber Layer Thickness (μm)	<0.001	0.25	0.63	0.54, 0.73	1.09

**Table 6:** Associations (Multivariable Analysis) between Retinal Vein Diameter and Ocular and Systemic Parameters including Estimated Cerebrospinal Fluid Pressure.

Characteristics	*P*-Value	Standardized Regression Coefficient beta	Non- Standardized Regression Coefficient B	95% Confidence Interval of B	Variance Inflation Factor
Estimated Cerebrospinal Fluid Pressure (mm Hg)	<0.001	0.09	0.78	0.47, 1.08	1.05
Gender	<0.001	-0.08	-3.98	-5.88,-2.09	1.06
Retinal Nerve Fiber Layer Thickness (μm)	0.03	0.22	0.56	0.46, 0.65	1.09
Retinal Arterial Diameter (μm)	<0.001	0.27	0.36	0.31, 0.41	1.08

## Discussion

In our community based study, thicker retinal artery diameter was associated with younger age, less abdominal circumference, lower diastolic blood pressure, thicker retinal nerve fiber layer and thinner common carotid artery IMT. The parameters with the strongest association were lower blood pressure and thicker retinal nerve fiber layer, while the association with common carotid artery IMT was relatively weak. In a reverse manner, thicker common artery IMT was associated with thinner retinal artery diameter after adjusting for older age, male gender, higher systolic blood pressure, lower level of education, higher prevalence of fatty liver disease, lower concentration of high-density lipoproteins and higher concentration of low-density lipoproteins. Thicker retinal vein diameter was associated with higher estimated cerebrospinal fluid pressure after adjusting male gender, thicker retinal nerve fiber layer thickness and wider retinal arteries. The strongest relationship was between wider retinal vein diameter and wider retinal arterial diameter and retinal nerve fiber layer thickness.

The results of our study agree with findings obtained in previous investigations. Wong and colleagues showed in their landmark study the association of retinal microvascular abnormalities, including retinal arteriolar narrowing, with incident stroke [1]. The latter has a thickening of the carotid arteries as one of the main risk factors. Applying magnetic resonance imaging of the brain, Hanff and associates found that retinal microvascular abnormalities predicted the progression of microvascular changes in the brain [2]. Torres and coworkers examined the association between the carotid IMT and retinal arteriolar and venular diameters in hypertensive patients [14]. In a cross-section study design, 173 hypertensive patients underwent fundus photography and carotid ultrasound for semi-automated carotid IMT measurement. The authors found a significant and independent association between carotid IMT and retinal arteriolar caliber (adjusted beta: -0.25; *P* = 0.001) and retinal venular caliber (adjusted beta: 0.19; *P* = 0.009) after controlling for age, gender, systolic blood pressure, total cholesterol and high-density lipoproteins, cholesterol, prior cardiovascular disease, carotid plaque and the retinal fellow vessel. Torres´ study and our investigation agree in the association between a thicker carotid artery IMT and thinner retinal arteries. They differ in the strength of the correlation. While in Torres´ study the association was relatively strong (adjusted beta: -0.25), the adjusted beta value in our study was considerably lower (beta: -0.06). Reasons for the discrepancy between the results of both studies may have been differences in the study population (arterial hypertensive patients versus population-based participant recruitment). Both studies also differed in the number of study participants, and in particular in the study design. In our study, thickness of the retinal nerve fiber layer was relatively strongly correlated with retinal arterial diameter while in Torres´ investigation the retinal nerve fiver layer thickness was not measured. The results of our study were in agreement with former ophthalmological studies in which optic nerve damage was strongly associated with a thinning of the retinal arteries [15,16]. This association also showed an intra-individual intraocular inter-hemispheric correlation in the sense that the retinal hemisphere with the more marked optic nerve damage showed the more pronounced reduction in retinal arterial caliber [17].

The retinal vein diameter was not significantly associated with the carotid artery IMT in multivariable analysis in our study population. This result cannot directly be compared with findings obtained in other studies since an association between venular diameter and atherosclerotic changes of the cerebroafferent arteries has not intensively been examined yet. Miller and colleagues examined the relationship between retinal vessel diameter and the incidence of coronary artery disease in 448 patients with type 1 diabetes [18]. After adjusting for the duration of diabetes, sex, arterial hypertension, serum lipids and smoking status, smaller arteriolar caliber was significantly associated with coronary artery disease while the venular caliber was not significantly associated. Taking coronary artery disease as a surrogate for carotid artery disease, the result of Miller´s study complies with the findings of our study, since both studies did not find a relationship between retinal venular diameter and atherosclerotic carotid artery changes. Kawasaki and colleagues reported in the Multi-Ethnic Study of Atherosclerosis that individuals with wider retinal veins (and narrower retinal arterioles) at baseline were more likely to develop arterial hypertension after adjusting for age, sex, race/ethnicity, arterial blood pressure and other vascular risk factors [19]. They concluded that wider retinal venular diameter (and narrower retinal arteriolar diameter) were associated with the development of hypertension independent of additional risk factors. It indirectly fit with the results of our study in which wider retinal vein thickness was associated with an increased estimated CSFP (or the combination of higher blood pressure, higher body mass index and younger age). In the Beaver Dam Eye Study, Myers and colleagues examined retinal venular diameter changes over time and found that the retinal vein diameter decreased with older age, a result which was in agreement with the findings of our investigation [20]. In contrast to our study, male gender was associated with wider retinal vein diameter.

Interestingly, thicker retinal vein diameter was associated with a higher estimated CSFP in our study after adjusting for wider retinal artery diameter, male gender and thicker retinal nerve fiber layer (Table 6). It agreed with previous population-based studies in which wider retinal vein diameter was also correlated with a higher estimated cerebrospinal fluid pressure, and in which a higher estimated cerebrospinal fluid pressure was correlated with a higher 10-year incidence of retinal vein occlusions [21]. As a corollary, a higher estimated CSFP was also associated with a thicker subfoveal choroid the veins of which, as the retinal veins, drain via the superior ophthalmic vein into the intracranial cavernous sinus [22]. Since the central retinal vein after leaving the eye pierces through the optic nerve and then passes through the orbital cerebrospinal fluid space before entering the retrobulbar orbital compartment, one may assume that the pressure in the central retinal vein should be at least as high as the orbital CSFP is. Correspondingly, the central retinal vein pressure as measured by ophthalmodynamometry correlated with CSFP in patients with increased brain pressure [8]. Fitting with the hypothesis of an association between retinal vein diameter and CSFP was the observation that retinal vein occlusions are usually noticed by patients in the morning after sleeping, during which the CSFP is increased due to hydrostatic reasons [23]. The pathogenic importance of the association between retinal vein diameter and CSFP may be explored in future studies.

Our study has potential limitations. Our study population was a randomly selected out of the population of the Kailuan Study, which, despite its large sample size was not fully representative for the Chinese population. Our study sample however was drawn from the Kailuan study population by using a stratified random sampling method based on the Chinese National Census from 2010. In that context, one may also discuss the "healthy worker effect", including the "healthy hire" and "healthy survivor" effect, that is present in many occupational cohort studies. It may be associated with a confounding effect. Second, our study was a cross-sectional investigation the study design of which did not allow drawing longitudinal conclusions on a causal relationship between retinal vessel diameter and atherosclerotic changes of the carotid arteries. Third, although we examined the retinal veins and explored associations between the retinal vein diameters and CSFP, we did not investigate the venous part of the neck vessels and we did thus not compare retinal vein measures with measurements of cerebrofugal veins. Future studies may address that topic.

In conclusion, thinner retinal artery diameter was significantly, however weakly, associated with increased common carotid artery IMT as a surrogate for atherosclerosis, after adjusting for optic nerve damage, younger age, higher blood pressure, and obesity. It suggested that retinal microvascular changes were only week indicators for the atherosclerosis of the carotid artery. Thicker retinal vein diameter was associated with higher estimated CSFP in multivariable analysis. Confirming previous studies on an association between higher estimated CSFP and higher incidence of retinal vein occlusion, it suggested an anatomical and functional relationship between renal vein diameter and brain pressure.
